# ﻿A new species of *Diglyphus* Walker (Hymenoptera, Eulophidae) from China, with morphological characterizations and molecular analysis

**DOI:** 10.3897/zookeys.1148.98853

**Published:** 2023-02-15

**Authors:** Wei-Jie Wan, Su-Jie Du, Christer Hansson, Wan-Xue Liu

**Affiliations:** 1 State Key Laboratory for Biology of Plant Diseases and Insect Pests, Institute of Plant Protection, Chinese Academy of Agricultural Sciences, Beijing, 100193, China Institute of Plant Protection, Chinese Academy of Agricultural Sciences Beijing China; 2 Biological Museum (Entomology), Lund University, Sölvegatan 37, SE-22362 Lund, Sweden Lund University Lund Sweden; 3 Natural History Museum, Life Sciences, Cromwell Road, London, UK Natural History Museum London United Kingdom

**Keywords:** 28S, Agromyzidae, biology, COI, ITS2, occurrence, parasitic wasp, phylogeny, taxonomy

## Abstract

*Diglyphus* Walker, 1844 (Hymenoptera: Eulophidae) is an economically important genus including species acting as biocontrol agents against agromyzid leafminer pests. A new species of *Diglyphus*, *Diglyphusdifasciatus* Liu, Hansson & Wan, **sp. nov.**, was discovered during the identification of agromyzid leafminers and their associated parasitoid wasps collected from 2016 to 2022 in China, based on morphological characteristics and molecular analyses of COI, ITS2 and 28S genes. *Diglyphusdifasciatus* is similar to *D.bimaculatus* Zhu, LaSalle & Huang, distinguished by two interconnected infuscate vertical bands on the fore wing and the color of the scape. Molecular data support *D.difasciatus* and *D.bimaculatus* as two different species. The mean genetic distances between *D.difasciatus* and *D.bimaculatus* were 11.33%, 8.62%, and 0.18%, based on the COI, ITS2, and 28S genes, respectively.

## ﻿Introduction

The genus *Diglyphus* (Hymenoptera: Eulophidae) was described by Walker (1844). Many taxonomists worldwide have summarized the typical *Diglyphus* morphological characteristics and introduced new *Diglyphus* species (Gordh and Hendrickson 1979; Zhu et al. 2000; Yefremova 2007; Hansson and Navone 2017). *Diglyphus* currently includes 41 species, and 17 of these are recorded from China (Gordh and Hendrickson 1979; Gauthier et al. 2000; Zhu et al. 2000; Liu et al. 2013; Hansson and Navone 2017; Ye et al. 2018; Noyes 2019).

*Diglyphus* is an economically important genus containing species that attack Agromyzidae (Diptera) leafminers and occasionally Lepidoptera pests (Gelechiidae, Gracillariidae, Lyonetiidae, and Nepticulidae) (Zhu et al. 2000; Yefremova et al. 2011; Hansson and Navone 2017; Noyes 2019). Agromyzidae leafminers such as *Chromatomyiahorticola* (Goureau) and *Liriomyza* spp. are pests of vegetables and ornamental plants worldwide (Bader et al. 2006; Kaspi and Parrella 2006; Foba et al. 2016; Rajender and Sharma 2016).

Identification of *Diglyphus* species mainly depends on morphological data. However, combining analyses of the morphology with molecular data for species identification is essential owing to the morphological similarities among species (Bernardo et al. 2008; Gebiola et al. 2012; Hansson and Navone 2017; Ye et al. 2018). The cytochrome *c* oxidase I (COI) gene of the mitochondrial DNA and internal transcribed spacer II (ITS2) ribosomal DNA genes have previously been applied to enhance species identification (Hebert et al. 2003; Bernardo et al. 2008; Gebiola et al. 2009; Gebiola et al. 2012; Gebiola et al. 2015; Du et al. 2021). Although 28S ribosomal DNA (28S) has mostly used for phylogenetic studies at the genus level and above, it has also been used for species identification (Gauthier et al. 2000; Gebiola et al. 2009; Burks et al. 2011; Gebiola et al. 2015).

For this project we collected *Diglyphus* material from 33 sites in China during 2016 to 2022 (Fig. 11). The specimens were reared mainly from the agromyzid *Chromatomyiahorticola* (Table 1). An undescribed species of *Diglyphus*, *D.difasciatus* Liu, Hansson & Wan, was discovered during the identification of the reared material. Altogether we recovered 125 female and 153 male specimens of *D.difasciatus* (Table 1).

**Table 1.** Collecting information of *Diglyphusdifasciatus* sp. nov. specimens.

Specimens	Sampling locality	GPS coordinates	Host plants	Host	Sampling date
5♀, 2♂ (4♀)	Longnan, Gansu	33°23'58"N, 104°49'39"E	*Sonchusoleraceus*	*C.horticola*	2019.05
1♀	Baiyin, Gansu	36°32'4"N, 104°10'21"E	*Phaseolusvulgaris*	*L.sativae*	2018.09
7♀, 1♂	Chifeng, Inner Mongolia	42°02'50"N, 120°23'25"E	*Phaseolusvulgaris*	Unknown	2018.08
1♂	Guyuan, Ningxia	36°01'31"N, 106°12'41"E	*Raphanussativus*	*C.horticola* and *L.huidobrensis*	2018.09
1♂	Guyuan, Ningxia	36°01'31"N, 106°12'41"E	*Sonchusoleraceus*	*C.horticola*	2018.09
1♀ (1♀)	Gonghe, Qinghai	36°16'35"N, 100°34'13"E	*Sonchusoleraceus*	*C.horticola*	2018.07
1♀ (1♀)	Baoji, Shaanxi	34°19'41"N, 107°13'56"E	*Chrysanthemummorifolium*	Unknown	2019.05
1♂	Baoji, Shaanxi	34°19'41"N, 107°13'56"E	*Glebioniscoronaria*	Unknown	2019.05
1♀, 1♂	Yantai, Shandong	37°17'26"N, 121°33'46"E	*Sonchusoleraceus*	Unknown	2017.05
1♀	Rizhao, Shandong	35°17'29"N, 119°11'37"E	*Phaseolusvulgaris*	*L.sativae*	2018.10
1♂ (1♂)	Linyi, Shandong	35°50'11"N, 118°28'56"E	*Brassicanapus*	*C.horticola*	2019.05
2♀, 4♂ (1♂)	Xinzhou, Shanxi	39°11'23"N, 113°15'14"E	*Lepidiumapetalum*	*C.horticola*	2017.06
1♀	Xinzhou, Shanxi	39°11'23"N, 113°15'14"E	*Alcearosea*	*C.horticola*	2017.06
1♀, 3♂	Xinzhou, Shanxi	39°11'23"N, 113°15'14"E	Brassicaceae sp.	*C.horticola* and *L.bryoniae*	2017.06
3♂ (2♂)	Linfen, Shanxi	36°04'30"N, 111°30'5"E	*Pisumsativum*	*C.horticola* and *L.trifolii*	2017.06
1♀, 2♂	Xinzhou, Shanxi	39°11'23"N, 113°15'14"E	*Lepidiumapetalum*	*C.horticola*	2017.06
3♂	Xinzhou, Shanxi	39°11'36"N, 113°16'27"E	*Sonchusoleraceus*	*C.horticola*	2017.07
1♀	Xinzhou, Shanxi	39°11'36"N, 113°16'27"E	Cirsiumarvensevar.integrifolium	*C.horticola* and *L.bryoniae*	2017.07
1♂	Xinzhou, Shanxi	39°11'36"N, 113°16'27"E	*Sonchusoleraceus*	Unknown	2017.07
6♀, 3♂ (3♀)	Xinzhou, Shanxi	39°11'36"N, 113°16'27"E	Asteraceae sp.	Unknown	2017.07
2♀, 2♂	Xinzhou, Shanxi	39°11'12"N, 113°14'30"E	*Lepidiumapetalum*	*C.horticola*	2018.05
2♀, 2♂ (1♀, 1♂)	Changzhi, Shanxi	36°11'8"N, 113°04'22"E	*Cirsiumjaponicum*	*C.horticola*	2018.05
1♀	Changzhi, Shanxi	36°11'8"N, 113°04'22"E	*Taraxacummongolicum*	*C.horticola*	2018.05
2♀, 2♂ (1♀, 1♂)	Yangquan, Shanxi	38°05'37"N, 113°22'45"E	*Alcearosea*	*C.horticola*	2018.05
3♂	Xinzhou, Shanxi	39°10'33"N, 113°17'35"E	Asteraceae sp.	*C.horticola*	2018.05
3♀ (2♀)	Xinzhou, Shanxi	39°10'33"N, 113°17'35"E	Asteraceae sp.	*C.horticola* and *L.sativae*	2018.09
3♀, 5♂ (2♀, 1♂)	Jincheng, Shanxi	35°29'33"N, 112°54'16"E	*Lepidiumapetalum*	*C.horticola*	2019.05
6♀, 12♂ (2♀, 3♂)	Jincheng, Shanxi	35°29'33"N, 112°54'16"E	*Crepidiastrumsonchifolium*	*C.horticola*	2019.05
5♀, 3♂ (2♀, 1♂)	Jincheng, Shanxi	35°29'33"N, 112°54'16"E	*Ixerispolycephala*	*C.horticola*	2019.05
2♀, 2♂ (1♂)	Beijing	40°01'22"N, 116°17'9"E	*Glebioniscoronaria*	*C.horticola*	2016.05
4♀, 23♂ (1♀,1♂)	Beijing	40°39'35"N, 117°13'55"E	*Raphanussativus*	*C.horticola*, *L.bryoniae* and *L.sativae*	2016.06
5♀, 3♂ (2♀, 1♂)	Beijing	40°39'35"N, 117°13'55"E	*Pisumsativum*	*C.horticola*	2016.06
2♀	Beijing	40°08'41"N, 116°45'36"E	*Glebioniscoronaria*	*C.horticola*	2017.05
1♂ (1♂)	Beijing	40°08'41"N, 116°45'36"E	*Glebioniscoronaria*	*C.horticola*	2017.05
2♂	Beijing	39°36'18"N, 116°18'57"E	*Ixerispolycephala*	*C.horticola* and *L.bryoniae*	2017.05
1♂	Beijing	40°01'17"N, 116°17'15"E	*Phaseolusvulgaris*	*C.horticola*	2017.08
5♀, 6♂	Beijing	40°01'34"N, 116°16'51"E	*Crepidiastrumsonchifolium*	*C.horticola*	2018.05
1♂ (1♂)	Beijing	40°16'21"N, 116°13'30"E	Lactucasativavar.asparagina	*C.horticola*	2018.05
4♀ (2♀)	Beijing	39°52'32"N, 116°11'21"E	*Ixerispolycephala*	*C.horticola*	2019.05
5♀ (3♀)	Beijing	39°36'18"N, 116°18'57"E	*Hemisteptialyrata*	*C.horticola* and *L.sativae*	2019.05
8♀, 14♂ (3♀, 3♂)	Beijing	40°01'23"N, 116°17'9"E	*Crepidiastrumsonchifolium*	*C.horticola*	2019.05
4♀, 2♂ (2♀, 1♂)	Beijing	40°01'23"N, 116°17'9"E	*Ixerispolycephala*	*C.horticola* and *L.bryoniae*	2019.05
1♂	Beijing	40°11'28"N, 116°28'0"E	*Luffaaegyptiaca*	Unknown	2019.08
2♀ (1♀)	Shijiazhuang, Hebei	37°51'27"N, 114°32'12"E	*Ixerispolycephala*	*C.horticola*	2017.05
3♀, 1♂ (2♀)	Shijiazhuang, Hebei	38°16'48"N, 114°41'59"E	Asteraceae sp.	*C.horticola*	2017.05
1♀ (1♀)	Shijiazhuang, Hebei	40°45'38"N, 114°51'32"E	*Lepidiumapetalum*	Unknown	2018.06
3♀, 5♂	Shijiazhuang, Hebei	41°09'11"N, 114°03'40"E	*Sonchusoleraceus*	*C.horticola*	2018.07
8♀, 10♂	Shijiazhuang, Hebei	41°09'11"N, 114°03'40"E	*Pisumsativum*	*C.horticola*	2018.07
1♀ (1♀)	Shijiazhuang, Hebei	41°09'11"N, 114°03'40"E	Lactucasativavar.asparagina	*C.horticola*	2018.07
6♀, 7♂	Shijiazhuang, Hebei	41°14'30"N, 114°09'25"E	*Lactucasativa*	*C.horticola* and *L.bryoniae*	2018.08
5♀, 14♂ (3♀, 4♂)	Zhangjiakou, Hebei	41°14'30"N, 114°09'25"E	*Lactucasativa*	*C.horticola*	2018.08
1♀ (1♀)	Zhangjiakou, Hebei	41°24'30"N, 114°09'8"E	*Taraxacummongolicum*	*C.horticola*	2019.07
2♂ (2♂)	Zhangjiakou, Hebei	41°24'30"N, 114°09'8"E	*Pisumsativum*	*C.horticola*	2019.07
1♀	Zhangjiakou, Hebei	41°24'30"N, 114°09'8"E	Asteraceae sp.	*C.horticola*	2019.08
2♀, 2♂	Zhangjiakou, Hebei	41°09'11"N, 114°03'40"E	*Lactucasativa* and Brassicarapavar.glabra	*C.horticola*	2022.08

Note: The number and sex of molecular identification specimens were in brackets.

## ﻿Materials and methods

### ﻿Sampling

We collected the leaves of vegetables and ornamental plants infested with agromyzid leafminers in different provinces of China from 2016 to 2022. The leaves were placed in cages and each cage was labeled with collection date, locality, and host plant. The collected leaf material was maintained in climate chambers set at 25 ± 1 °C, 30–50% relative humidity, and a photoperiod of 14:10 h (light: dark) until agromyzid leafminers and their parasitoids emerged. All wasp specimens and their hosts were preserved in absolute ethanol and maintained at -20 °C at the Institute of Plant Protection (**IPP**), Chinese Academy of Agricultural Sciences (**CAAS**), Beijing, China. All data for *D.difasciatus* specimens are presented in Table 1.

Two males and two females of *D.difasciatus* reared from *C.horticola* were imaged and morphologically characterized. One male and one female (the holotype) were reared from leaves of *Lactucasativa* Linn. and Brassicarapavar.glabra Regel in Hebei, China; one female was reared from leaves of *Sonchusoleraceus* Regel in Gansu, China; one male was reared from leaves of *L.sativa* in Hebei, China. Two female and one male of *D.bimaculatus* Zhu, LaSalle & Huang were used for imaging and morphological characterization, which were reared from leaves of *Sonchusoleraceus* in Tibet, China. Specimens used for molecular analyses included 67 specimens of *D.difasciatus*, 1♀ *D.bimaculatus* (Tibet), and 1♀ *D.isaea* (Walker) (Hubei). *Diglyphusbimaculatus* and *D.isaea* sequences were used as outgroups for analyzing the phylogenetic relationship of *D.difasciatus.* Furthermore, one female of *D.bimaculatus* used for molecular analyses was reared from leaves of *Taraxacummongolicum* Hand.-Mazz., which was collected from Tibet, China (29°39'3"N, 91°08'41"E) in August 2020. The single *D.isaea* specimen was reared from *C.horticola* in *Pisumsativum* Linn. leaves, collected in Hubei, China (30°28'26"N, 114°21'17"E) in April 2017.

### ﻿Morphological identification methods

The specimens were examined using a stereomicroscope (Olympus, SZX-16). Photographs were taken using an Olympus BX43 microscope equipped with a Helicon Focus 6.

The morphological terminology and measurement methods follow Gibson (1989), Hansson (1990), and Yefremova et al. (2011), and the following abbreviations were used:

**F1–2** Flagellomeres 1–2: maximum length of flagellomeres 1–2.

**OOL** Ocular ocellar line: shortest distance between the lateral ocelli and eyes.

**POL** Posterior ocellar line: shortest distance between lateral ocelli.

### ﻿Molecular diagnosis methods

Genomic DNA was extracted from the metasoma of each specimen. The extraction methods followed those described by De Barro and Driver (1997), with some modifications. The DNA extraction was performed using a 200-µL microcentrifuge tube (Bioevopeak, Shandong, China) and 200-µL pipette tip (Bioevopeak) sealed by heating to grind the metasoma into a homogenate. The homogenate was incubated at 65 °C, 25 °C, and 96 °C for 30, 2, and 10 min, respectively. After extraction, the genomic DNA was stored at -20 °C until molecular diagnosis. The primers used for amplification were in Table 2.

**Table 2.** Primers used for amplification.

Gene	Primers	Sequences (5’-3’)	References
COI	LCO1490	GGTCAACAAATCATAAAGATATTGG	Folmer et al. 1994
	HCO2198	TAAACTTCAGGGTGACCAAAAAATCA	Folmer et al. 1994
ITS2	ITS2F	TGTGAACTGCAGGACACATG	Campbell et al. 1993
	ITS2R	AATGCTTAAATTTAGGGGGTA	Campbell et al. 1993
28S	D2-3549F	AGTCGTGTTGCTTGATAGTGCAG	Campbell et al. 1993
	D2-4068R	TTGGTCCGTGTTTCAAGACGGG	Campbell et al. 1993

Amplifications were performed as described by Hebert et al. (2003) and Du et al. (2021). Polymerase chain reaction (PCR) consisted of 0.4 μL *Taq* enzyme (2.5 UμL^-1^), 0.4 μL deoxynucleotide triphosphate (2.5 mM), 2.5 μL 10× buffer (containing Mg^2+^), 0.4 μL forward primer, 0.4 μL reverse primer, 1 μL DNA template, and 19.9 μL double-distilled H_2_O. Next, the PCR cycling conditions consisted of an initial denaturation at 95 °C for 5 min, followed by 35 cycles of denaturation at 95 °C for 30 s, annealing for 45 s, extension at 72 °C for 60 s, and a single cycle of final extension at 72 °C for 5 min. The annealing temperatures for the COI, ITS2, and 28S genes were 50 °C, 52 °C, and 58 °C, respectively.

The unpurified PCR products were sent to Sangon Biotech Co., Ltd, Beijing, China, for bidirectional sequencing, and primers were designed by Sangon Biotech Co., Ltd, Beijing, China. The PCR instrument used was an ABI thermal cycler (Applied Biosystems Veriti 9902; Woburn, MA, USA).

### ﻿Sequence analysis

The *D.difasciatus* sequences were analyzed using the National Center for Biotechnology Information (NCBI, https://www.ncbi.nlm.nih.gov/) and the Barcode of Life Data systems (BOLD, http://www.boldsystems.org/index.php). The phylogenetic relationships between *D.difasciatus*, *D.bimaculatus*, and *D.isaea* were also analyzed.

All sequences were aligned following the default options of the CLUSTAL W tool (Kimura 1980); in Molecular Evolutionary Genetics Analysis (MEGA) X ver. 10.1.8 (Kimura 1980; Kumar et al. 2018). Pairwise and mean sequence divergence were estimated based on the Kimura-2 parameter (K2-P) (Kimura 1980). Gene haplotypes were calculated using DNA sequence polymorphism ver. 5 (Bioinformatics, Arlington, VA, USA) (Librado and Rozas 2009). The phylogenetic tree was constructed using the Neighbor-Joining method in MEGA X (Kimura 1980; Nei and Kumar 2000; Kumar et al. 2018). Bootstrap values were obtained after 1000 replications for sequence divergence and phylogenetic relationships. Bootstrap support values ≥75% is indicated above the branches of the phylogenetic tree.

## ﻿Results

### ﻿Taxonomy

#### ﻿ Diglyphus difasciatus

Taxon classificationAnimaliaHymenopteraEulophidae

Liu, Hansson & Wan sp. nov.

0DC03C5B-460A-5818-9FC7-E908C5F6539B

https://zoobank.org/DA756718-6C6B-4958-8375-BED1BF06EE3C

Figs 1–2 , 3–7

##### Material.

***Holotype* female**: China, Hebei; 41°09'11"N, 114°03'40"E; 25 August 2022; Miao-Miao Mao leg.; reared from *Chromatomyiahorticola* on leaves of *Lactucasativa* and Brassicarapavar.glabra, deposited in IPP. ***Paratypes***: 1♀ 2♂ with same label data as holotype, deposited in National Animal Collection Resource Center, Institute of Zoology, Chinese Academy of Sciences. 1♀ China, Beijing; 39°52'32"N, 116°11'21"E; 11 May 2019; Qiang Wu leg.; reared from *C.horticola* on leaves of *Sonchusoleraceus* and *Ixerispolycephala*, deposited in National Animal Collection Resource Center, Institute of Zoology, Chinese Academy of Sciences. 2♀ China, Beijing; 39°36'18"N, 116°18'57"E; 20 May 2019; Jing He and Meng Guo leg.; reared from *C.horticola* on leaves of *Hemisteptialyrata*, deposited in IPP. 1♀ 2♂ China, Shanxi; 39°11'23"N, 113°15'14"E; 6 June 2017; Zhu-Sheng Zheng leg.; reared from *C.horticola* on leaves of *Lepidiumapetalum*, deposited in IPP. 1♀ 2♂ China, Shanxi; 36°11'8"N, 113°04'22"E; 17 May 2018; Jing He and Su-Jie Du leg.; reared from *C.horticola* on leaves of *Cirsiumjaponicum*, deposited in IPP. 3♀ 5♂ China, Shanxi; 35°29'33"N, 112°54'16"E; 9 May 2019; Jing He and Su-Jie Du leg.; reared from *C.horticola* on leaves of *Crepidiastrumsonchifolium*, deposited in IPP. 2♀ 1♂ China, Hebei; 38°16'48"N, 114°41'59"E; 14 May 2017; Rong-Jun Zhen and Gui-Fen Zhang leg.; reared from *C.horticola* on leaves of an unidentified Asteraceae, deposited in IPP.

##### Diagnosis.

**Figures 1–2.**
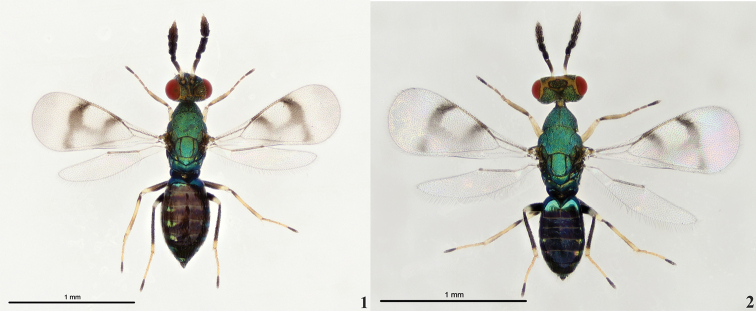
*Diglyphusdifasciatus* sp. nov. **1** female holotype, habitus, dorsal view **2** male paratype, habitus, dorsal view.

**Figures 3–8.**
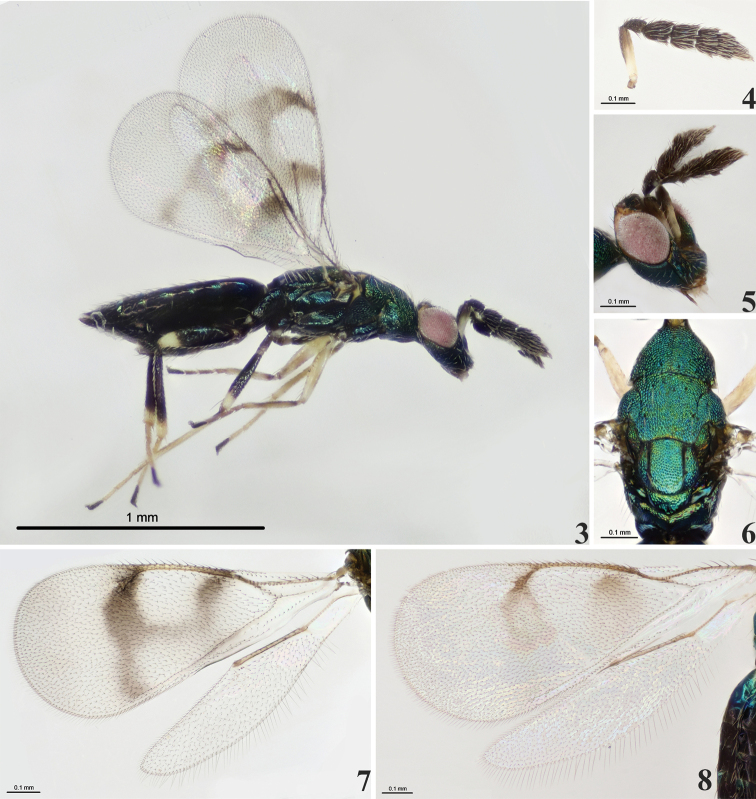
*Diglyphus* spp. **3–7***D.difasciatus* sp. nov. **3** female paratype, habitus, lateral view **4** female paratype, antenna, lateral view **5** male paratype, head, lateral view **6** male paratype, mesosoma, dorsal view **7** female holotype, left fore and hind wings, dorsal view **8***D.bimaculatus* Zhu, LaSalle & Huang, female, left fore and hind wings, dorsal view.

Scape white with apical 1/3–1/2 dark brown (Figs 1–5). The yellow markings on the vertex and face, and those on the male are wider than those on the female. Fore wing with complete vertical infuscate bands below base of marginal and stigmal veins respectively, the two bands are interconnected medially (Figs 1–3, 7); speculum bare, without dense setae and postmarginal vein almost equal in length to stigmal vein (Figs 1–3, 7). Mid and hind femora black with apical 1/4 yellowish-white (Figs 1–3). Fore and mid tibia yellowish-white with a dark ring basally (Figs 1–3). Hind tibia black with apical 1/5 yellowish-white (Figs 1–3). Pretarsus on all legs black (Figs 1–3).

##### Description.

Female (Fig. 1). Body length 1.6mm, Fore wing length 0.8mm. Scape white with apical 1/3–1/2 dark brown. Pedicel and flagellum dark brown. Head dark brown. Eyes red and ocelli brown. Mandibles brownish. Yellow markings on the vertex and face. Pronotum, mesoscutum, scutellum, dorsellum, and propodeum metallic blue-green. Fore wing with two complete vertical infuscate bands below base of marginal and stigmal veins respectively, the two bands are interconnected medially (Fig. 7). Mid and hind femora black with apical 1/4 yellowish-white. Fore and mid tibia yellowish-white with a dark ring basally. Hind tibia black with apical 1/5 yellowish-white. Gaster dark brown.

***Head*** (Fig. 1). Head length 0.6× width in dorsal view, and length 0.9× width in frontal view. POL 1.8× OOL. Malar space 0.7× height of eye, and malar sulcus present. Frons and vertex with distinct reticulation. Eyes with sparse and short setae. Toruli situated below the level of lower margin of eyes. Maxillary palpus with two segments and labial palpus with one segment. Antennal flagellum with two funiculars and three clavomeres; scape 4.0× as long as broad and 2.8× as long as pedicel; pedicel 1.3× as long as broad; F1 1.4× and F2 0.9× as long as broad, F1 1.5× as long as F2; clava 2.4× as long as broad, 1.3× as long as scape, and 3.6× as long as F2.

***Mesosoma*** (Fig. 1). Pronotum without transverse carina, reticulate, shorter than mesoscutum. Mesoscutum 1.2× as long as scutellum; mid lobe with two pairs of long setae; notauli incomplete and diverging posteriorly to meet anterior part of axillae. Setae on pronotum and mesoscutum pale. Scutellum as long as broad with straight sublateral grooves and two pairs of setae. Dorsellum with superficial reticulation with isodiametric meshes, posterior margin round. Propodeum shorter than scutellum and without median carina; callus with five setae. Fore wing with 5–7 setae on dorsal surface of submarginal vein; speculum mainly bare, with few scattered setae; costal cell with two rows of setae, including 15 setae at the base of costal cell and an incomplete row with eight setae in apical part; postmarginal vein almost equal in length to stigmal vein; Fore wing length 1.7× fore wing width. Petiole short and inconspicuous. Gaster subrotund, 1.9× as long as wide in dorsal view; apex acute. Tip of ovipositor sheaths visible in dorsal view.

**Male** (Fig. 2). Similar to the female. Body length 1.4mm, Fore wing length 0.8mm. Head length 0.5× width in dorsal view, and length 0.8× width in frontal view. POL 1.1× OOL. Scape 4.7× as long as broad, 2.2× as long as pedicel. Pedicel 1.6× as long as broad. Antennal flagellum with two funiculars and three clavomeres, F1 0.8× and F2 0.7× as long as broad, F1 1.2× as long as F2. Clava 3.1× as long as broad, 1.1× as long as scape and 4.6× as long as F2. Mesoscutum 1.2× as long as scutellum. Scutellum as long as broad. Fore wing length 1.7× as long as fore wing width. Gaster 1.8× as long as wide in dorsal view.

##### Variation.

Females are slightly larger than males (1.6 mm and 1.4 mm, respectively).

##### Hosts and biology.

*Diglyphusdifasciatus* is a larval ectoparasitoid, primarily on *Chromatomyiahorticola*, and occasionally on *Liriomyzabryoniae* (Kaltenbach), *L.sativae*, and *L.trifolii* (Burgess). The hosts are usually mining in leaves of Asteraceae, Brassicaceae and Fabaceae, especially on *Ixerispolycephala* Cass. ex DC. and *Pisumsativum* (Table 1). *Diglyphusdifasciatus* occurs and reaches its highest occurrence period in May, and then disappears in October. Female *Diglyphus* exhibit three types of host-killing behavior (Zhu et al. 2000; Liu et al. 2013; Hansson and Navone 2017; Ye et al. 2018). The host-killing behavior of *D.difasciatus* is not known and requires further studies.

##### Distribution.

China (Beijing, Gansu, Hebei, Inner Mongolia, Ningxia, Qinghai, Shaanxi, Shandong, and Shanxi).

##### Etymology.

The name is derived from a combination of the Latin *di* (double) and *fascia* (band) by referring to the two vertical infuscate bands in the fore wings.

##### Comments.

**Figures 9–10.**
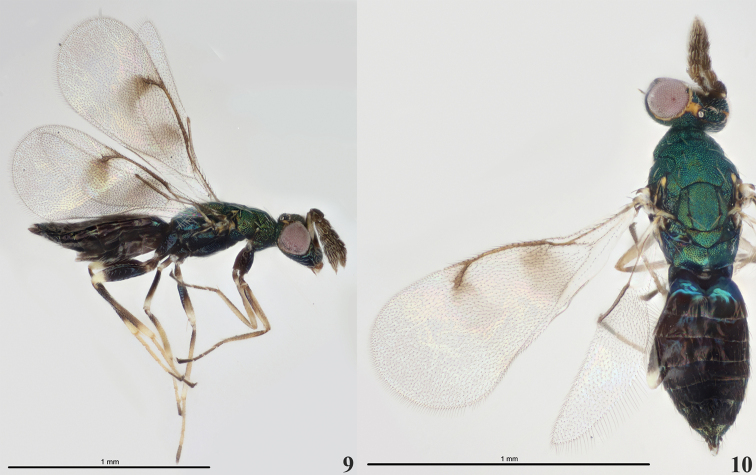
*Diglyphusbimaculatus***9** female habitus, lateral view **10** male habitus, dorsal view.

*Diglyphusdifasciatus* is very similar to *D.bimaculatus* (Figs 8–10), but has two complete vertical infuscate bands that are interconnected medially in the fore wing, whereas *D.bimaculatus* has two infuscate spots in the fore wing. In addition, the scape of *D.difasciatus* is white with apical 1/3–1/2 dark brown (Figs 1–5), which is less than the scape of *D.bimaculatus* with white upper surface (Fig. 9). Besides, molecular data support the separation of these two morphologically similar species as distinct species.

### ﻿Molecular identification results

#### COI

The length of COI sequences from 67 *D.difasciatus* specimens was 514 bp, including 35 variable sites with 20 parsimony-informative sites, and 29 haplotypes were found (Fig. 12). The highest percentage similarity (89%) of sequences between *D.difasciatus* and other *Diglyphus* species in the NCBI and BOLD databases was with *D.pulchripes* (Erdös & Novicky) (NCBI accession number: MG442711).

**Figure 11.**
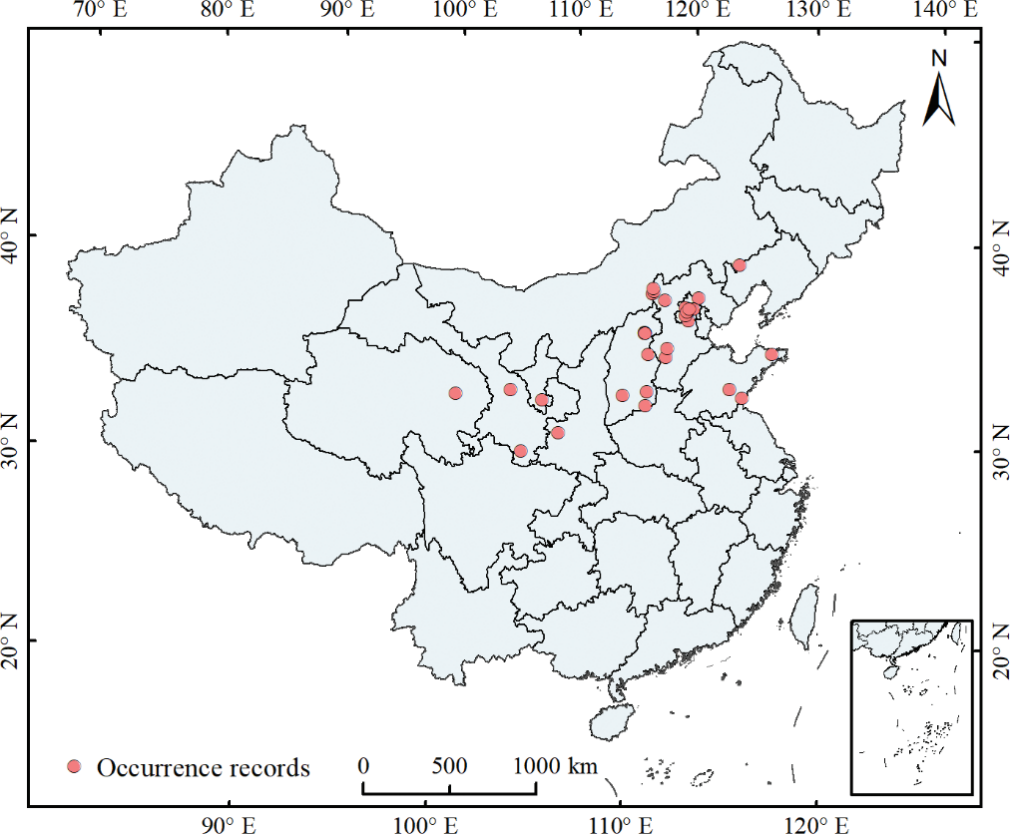
Collection sites of *Diglyphusdifasciatus* sp. nov. in China from 2016 to 2022.

**Figure 12.**
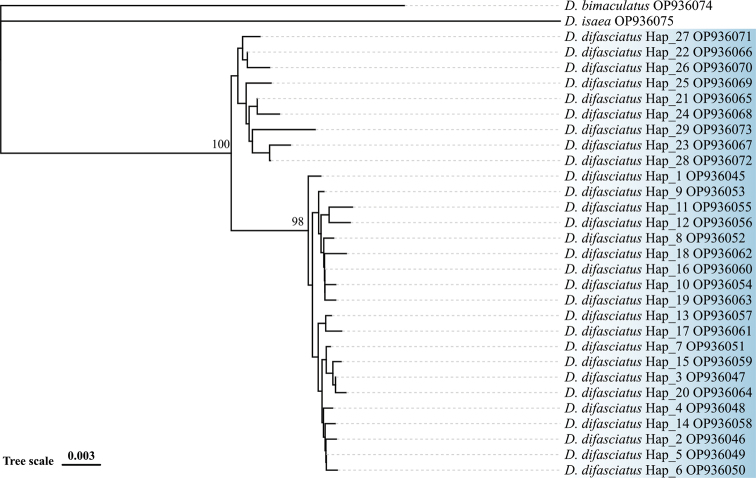
Phylogenetic tree of the three *Diglyphus* species based on the COI gene. The phylogenetic tree was constructed using the maximum likelihood method based on the Neighbor-Joining model. Accession numbers submitted to GenBank are shown next to each haplotype, and bootstrap support values (≥ 75%) are shown next to the branches.

The mean genetic distance of COI sequences between *D.difasciatus*/*D.bimaculatus* and *D.difasciatus*/*D.isaea*, based on the COI gene, was 11.33% and 13.37%, respectively (Table 3). The analyses of the intraspecific diversity in *D.difasciatus* showed that the mean genetic distance between the 29 haplotypes was 1.53% (Table 3). The genetic distance within *D.difasciatus* ranged from 0.19 to 3.42%.

**Table 3.** The mean genetic distance between three *Diglyphus* species based on the COI, ITS2 and 28S genes.

	Species	COI			ITS2			28S		
		1	2	3	1	2	3	1	2	3
1	*D.difasciatus*	0.0153			–			–		
2	*D.bimaculatus*	0.1133	–		0.0862	–		0.0018	–	
3	*D.isaea*	0.1337	0.1485	–	0.0649	0.0414	–	0.0018	0.0037	–

#### ITS2

The length of the 25 *D.difasciatus* sequences was 415 bp; there were no variable sites. The highest percentage similarity of sequences in the NCBI and BOLD databases was between *D.difasciatus* and *D.isaea* (86%). The mean interspecific genetic distance between *D.difasciatus*/*D.bimaculatus* and *D.difasciatus*/*D.isaea* was 8.62% and 6.49%, respectively (Table 3).

#### 28S

The length of the 11 sequences obtained from *D.difasciatus* was 547 bp; there were no variable sites. The highest percentage similarity of sequence in NCBI and BOLD between *D.difasciatus* and other *Diglyphus* species was with *D.crassinervis* (100% [NCBI accession number: MW393686]). The mean interspecific genetic distance between *D.difasciatus*, *D.bimaculatus* and *D.isaea* was 0.18% (Table 3).

All gene sequences are uploaded to GenBank with accession numbers OP933727–OP933732 and OP936054–OP936075.

## ﻿Discussion

The new species, *D.difasciatus*, is defined by morphological data and molecular data from the genes COI, ITS2, and 28S. Morphologically, *D.difasciatus* is most similar to *D.bimaculatus*, from which it can be separated by a different wing pattern in the fore wing and the color of scape (Figs 3, 7–9). Molecular data from COI, ITS2, and 28S also show that *D.difasciatus* and *D.bimaculatus* are two different species.

## Supplementary Material

XML Treatment for Diglyphus difasciatus

## Supplementary materials

Supplementary material 1 The genetic distance between three *Diglyphus* species based on the COI gene Data type phylogenetic https://binary.pensoft.net/file/810486This dataset is made available under the Open Database License (http://opendatacommons.org/licenses/odbl/1.0/). The Open Database License (ODbL) is a license agreement intended to allow users to freely share, modify, and use this Dataset while maintaining this same freedom for others, provided that the original source and author(s) are credited.Wei-Jie Wan, Su-Jie Du, Christer Hansson, Wan-Xue Liu
